# A novel role for the chloride intracellular channel protein Clic5 in ciliary function

**DOI:** 10.1038/s41598-023-44235-y

**Published:** 2023-10-17

**Authors:** Elisabeth Ott, Sylvia Hoff, Lara Indorf, Franck Anicet Ditengou, Julius Müller, Gina Renschler, Soeren S. Lienkamp, Albrecht Kramer-Zucker, Carsten Bergmann, Daniel Epting

**Affiliations:** 1 Department of Medicine IV, Faculty of Medicine, Medical Center-University of Freiburg, 79106 Freiburg, Germany; 2 Bio Imaging Core Light Microscopy (BiMiC), Medical Faculty-Institute for Disease Modeling and Targeted Medicine (IMITATE), 79106 Freiburg, Germany; 3 Limbach Genetics, Medizinische Genetik Mainz, 55128 Mainz, Germany; 4 Center for Biological Signaling Studies (BIOSS), 79104 Freiburg, Germany

**Keywords:** Developmental biology, Genetics, Molecular biology

## Abstract

CLIC5 belongs to a family of ion channels with six members reported so far. In vertebrates, the *CLIC5* gene encodes two different isoforms, CLIC5A and CLIC5B. In addition to its ion channel activity, there is evidence for further functions of CLIC5A, such as the remodeling of the actin cytoskeleton during the formation of a functional glomerulus in the vertebrate kidney. However, its specific role is still incompletely understood and a specific functional role for CLIC5B has not been described yet. Here we report our findings on the differential expression and functions of Clic5a and Clic5b during zebrafish kidney development. Whole-mount in situ hybridization studies revealed specific expression of *clic5a* in the eye and pronephric glomerulus, and *clic5b* is expressed in the gut, liver and the pronephric tubules. Clic5 immunostainings revealed that Clic5b is localized in the cilia. Whereas knockdown of Clic5a resulted in leakiness of the glomerular filtration barrier, Clic5b deficient embryos displayed defective ciliogenesis, leading to ciliopathy-associated phenotypes such as ventral body curvature, otolith deposition defects, altered left–right asymmetry and formation of hydrocephalus and pronephric cysts. In addition, Clic5 deficiency resulted in dysregulation of cilia-dependent Wnt signalling pathway components. Mechanistically, we identified a Clic5-dependent activation of the membrane-cytoskeletal linker proteins Ezrin/Radixin/Moesin (ERM) in the pronephric tubules of zebrafish. In conclusion, our in vivo data demonstrates a novel role for Clic5 in regulating essential ciliary functions and identified Clic5 as a positive regulator of ERM phosphorylation.

## Introduction

Cilia are antennae-like vestigial organelles that project from the outside of virtually all eukaryotic cells. Primary cilia are typically non-motile, sense extracellular signals, and act as regulators of signalling pathways that are important for development and maintenance of tissue homeostasis. Motile cilia beat in a wave-like pattern, leading to a fluid movement, for example to clear the mucus in the trachea. Defects in ciliary assembly or function lead to a wide range of disease symptoms collectively referred as ciliopathies^1–4^. The six chloride intracellular channel (CLIC) members, CLIC1-6, constitute a distinct family of chloride channels that are found on cell membrane, and also occur as cytoskeletal-associated and soluble cytosolic forms^5–8^. The first identified and characterized mammalian CLIC protein was p64 that was isolated from bovine kidney^9–12^. All CLICs share a highly conserved CLIC core homology domain while their N-terminal region varies considerably in amino acid (aa) sequence and length^13–15^. CLIC5 consists of two isoforms, CLIC5A (251aa in humans; 32 kDa) and CLIC5B (410aa in humans; 49 kDa), which is a result of alternative splicing of their first exons. CLIC5 (CLIC5A) was originally isolated from human placental microvilli, and identified as a component of a cytoskeletal multiprotein complex^13^. First in vitro studies demonstrated that CLIC5A is localized in phospholipid membranes, and revealed chloride-selective channel activity that has been reported thus far only for the CLIC member CLIC1^16^. Following studies demonstrated poorly selective rather than chloride-selective channel activity for CLIC1, CLIC4 and CLIC5 in vitro. Moreover, these studies revealed actin-regulated channel activity for CLIC1 and CLIC5 but not for CLIC4^17,18^. In addition, in vitro chloride channel activity was recently identified for CLIC2 and CLIC3^19,20^. However, the association of CLIC proteins with ion channel activity remains elusive, as it is based on artificial membrane conditions and to date there is no substantiate evidence for it under physiological conditions^8^. The longer CLIC5 isoform, CLIC5B, may be the human orthologue of bovine p64^21^. Apart from its classification as a member of the CLIC family and its confirmed chloride channel activity in vitro, the association with various cytoskeletal proteins strongly suggested additional functions for CLIC5. Analysis of the *Clic5* mutant mouse jitterbug (*jbg*), revealed dysmorphic stereocilia, and in the inner ear progressive hair cell degeneration that leads to hearing loss and vestibular dysfunction. These phenotypes have been described also in Radixin deficient mice^22^. In the same report, CLIC5 immunostainings showed a similar localization as the ERM (Ezrin, Radixin, Moesin) protein Radixin at the base of the stereocilia. It has also been reported that CLIC5 is expressed at high levels in the endothelium and podocytes of renal glomeruli in the mouse^23,24^. CLIC5 is co-localized and associated with ERM proteins and Podocalyxin in the glomerular podocytes, and thereby plays an important role in the development or maintenance of a functional glomerular filtration barrier, or both of these, in vivo. Both CLIC5 isoforms are expressed in the glomeruli and CLIC5 deficiency leads to reduced levels of Ezrin, phosphorylated ERM and Podocalyxin resulting in loss of podocyte integrity and subsequently to proteinuria in the *jbg* mutant mice^23^. Mechanistically, CLIC5A has been shown to activate the small GTPase Rac1 which in turn is required for CLIC5A mediated 4,5-bisphosphate (PI(4,5)P2)-dependent regulation of Ezrin activity^25–27^. To date, two reports present *CLIC5* mutations in individuals diagnosed with autosomal recessive non-syndromic hearing impairment (arNSHI) that show close similarities to the phenotypes observed in the *jbg* mouse mutant^28,29^. The first report identified a homozygous nonsense mutation in *CLIC5* (c.96T > A; p.(Cys32Ter)) in two affected siblings of a consanguineous Turkish family, and both had progressive hearing impairment, vestibular dysfunction and a mild renal dysfunction^28^. The second report identified bi-allelic compound heterozygous pathogenic variants in *CLIC5* (c.224T > C; p.(L75P) and c.63 + 1G > A) in a Cameroonian multiplex family with three affected individuals with hearing impairment^29^.

In this study, we analysed in detail the role of Clic5 in the vertebrate model zebrafish. We demonstrate that Clic5a deficiency results in leakiness of the glomerular filtration barrier in zebrafish. Interestingly, we identified a novel cilia-related role for Clic5 by showing that Clic5b deficiency leads to a wide range of well-known ciliopathy-associated phenotypes. Furthermore, we show that Clic5b acts as a positive regulator of ERM activity.

## Results

### Identification of zebrafish orthologues of human CLIC5

The Ensembl genome browser was used to identify a putative zebrafish orthologue of human *CLIC5*. This search resulted in the identification of two zebrafish *clic5* genes mapped to chromosome 17 (ENSDARG00000075993) and chromosome 20 (ENSDARG00000070584), the latter one with two isoforms (ENSDART00000100571 and ENSDART00000037379). Sequence analysis revealed an open reading frame of 741 bp for ENSDARG00000075993, 732 bp for ENSDART00000100571 and 1227 bp for ENSDART00000037379 encoding proteins. These have respectively 246 (Accession: NP_001007386), 243 (Accession: NP_001091668) and 408 (Accession: NP_998062) amino acids. Homology screening, with the human CLIC5A amino acid sequence as a query, revealed 79% identity to NP_001007386 and NP_001091668. We termed NP_001091668 zebrafish Clic5a and NP_001007386 (that most likely represents a Clic5a paralogue) zebrafish Clic5al. The N-terminal sequences of known mammalian CLIC5B proteins vary considerably in length and sequence. Amino acid alignment of solely the N-terminal parts from the longer isoform (NP_998062) and mammalian CLIC5B proteins revealed 24–27% homology, and was therefore termed zebrafish Clic5b. Phylogenetic analysis of vertebrate CLIC1-5 family members revealed highest conservation of the two zebrafish Clic5 isoforms to the CLIC4 and CLIC5 of *Xenopus*, mouse and human (Suppl. Fig. 1A). The Clic5 core domain of zebrafish Clic5 shares high conservation to the CLIC5 core domain of different species (Suppl. Fig. 1B).

### Expression of *clic5a* and *clic5b* during zebrafish development

**Figure 1:**
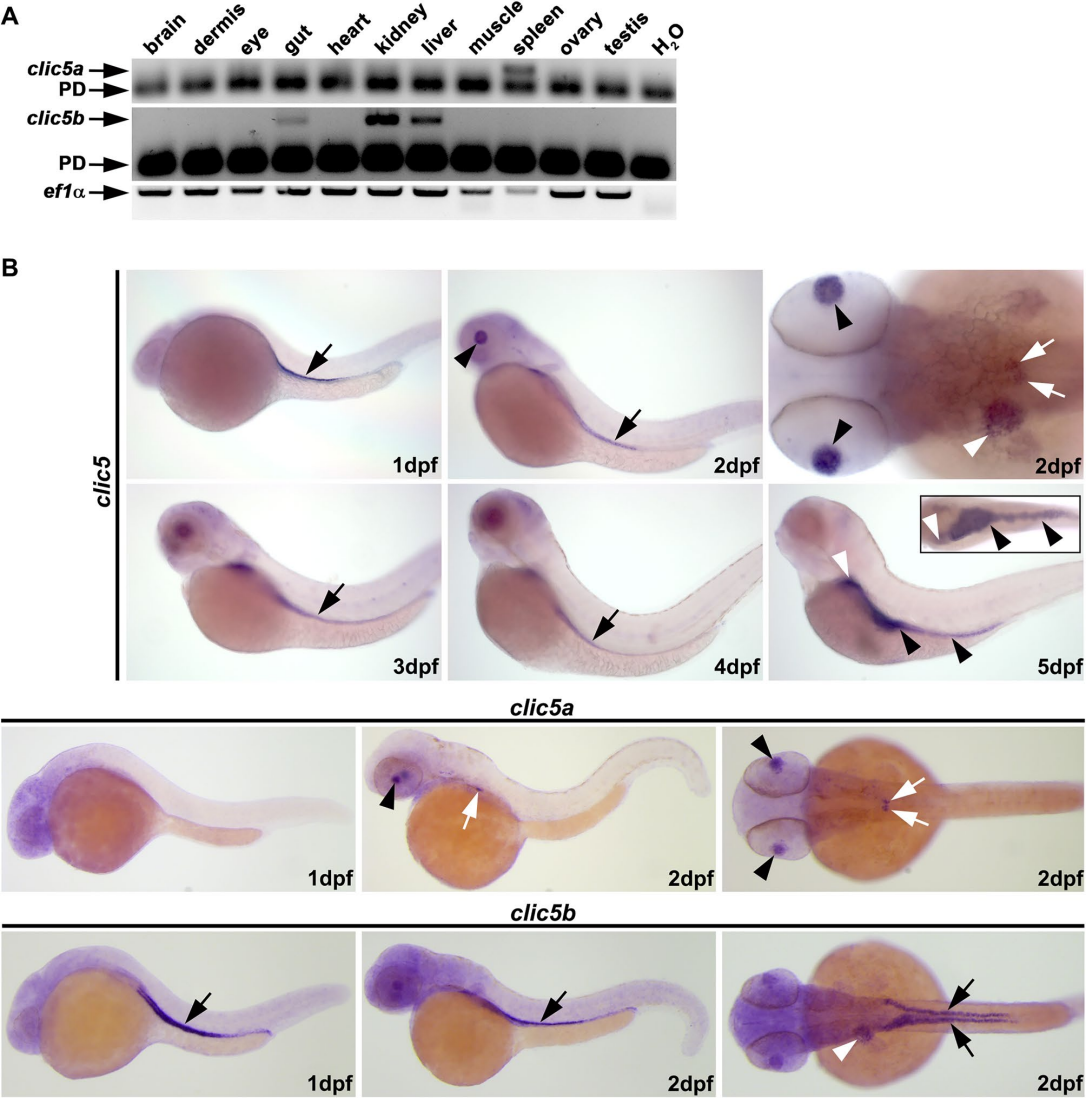
Expression analyses of *clic5* in zebrafish. **(A)** Semi-quantitative reverse-transcription polymerase chain reaction (RT-PCR) on cDNA of different adult zebrafish organs revealed expression of *clic5a* (223 bp) in the spleen and of *clic5b* (408 bp) in the gut, kidney and liver. H_2_O served as negative control and *ef1α* as loading control; Primer dimer (PD). **(B)** Whole-mount in situ hybridization (WISH) analysis reveals expression of *clic5* in the pronephric tubules (black arrow) at 1 day post-fertilization (dpf) which persisted throughout the first days of zebrafish development. At 2dpf, *clic5* was additionally expressed in the eye lenses (black arrowheads), liver primordium (white arrowhead), pectoral fin buds and glomerulus (white arrows). At 5dpf *clic5* expression was detectable in the liver (white arrowhead) and gut (black arrowheads) (framed box shows the expression of *clic5* in the liver and gut from the dorsal view). WISH analysis with specific probes for *clic5a* and *clic5b* reveals expression of *clic5a* in the eye lenses and glomerulus at 2dpf and expression of *clic5b* in the pronephric tubules at 1 and 2dpf with additional expression in the pectoral fin buds and liver primordium at 2dpf.

**Figure 2:**
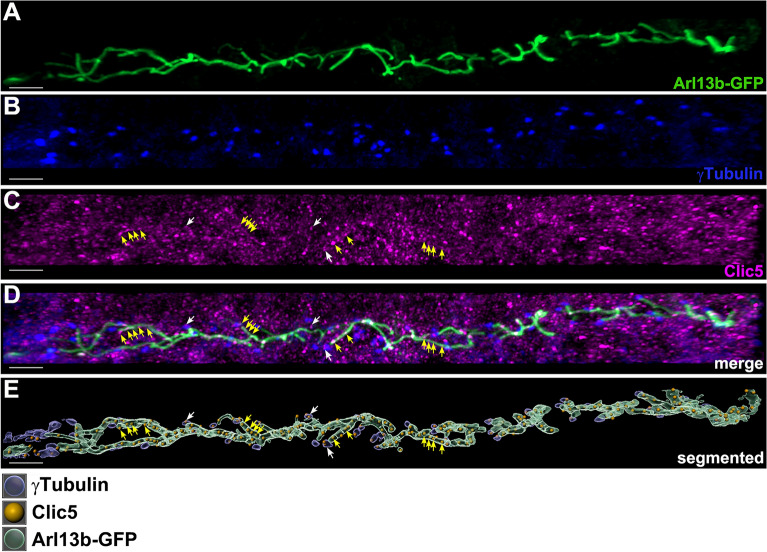
Subcellular localization analyses of Clic5 in the pronephric tubule of zebrafish. **(A–D)** Triple whole-mount immunostaining on 1dpf old *Tg(actb2:Mmu.Arl13b-GFP)* zebrafish embryos using anti-GFP (labeling Arl13b-GFP, i.e. the ciliary axoneme) **(A)**, anti-γTubulin (labeling the ciliary basal body) **(B)** and anti-Clic5 (monoclonal antibody produced in mouse) **(C)** antibodies reveals ciliary localization of Clic5 in the pronephric tubule. **(D)** Image is **(A–C)** merged. **(E)** 3D reconstruction and segmentation of Arl13b-GFP (green), γTubulin (blue) and Clic5 (gold) using Imaris software. Clic5 voxels co-localize with those of ciliary basal body (white arrows) and ciliary axoneme voxels (yellow arrows), respectively. Scale bar, 5 µm.

To determine the temporal and spatial expression pattern of *clic5* isoforms in zebrafish, we performed semi-quantitative RT-PCR on cDNA of different early developmental stages and adult organs, and also whole-mount in situ hybridization (WISH) studies. RT-PCR analyses revealed that *clic5a* and *clic5b* are hardly detectable during early zebrafish development. Whereas *clic5a* shows from all analysed organs expression only in the spleen, *clic5b* transcripts are detectable in the gut, kidney and liver (Fig. 1A). WISH analyses revealed that there was no detectable expression for *clic5al*, and that *clic5a* and *clic5b* showed a specific but distinct expression during zebrafish development (Fig. 1B, Suppl. Fig. 2). Using a pan WISH probe revealed specific expression of *clic5* in the pronephric tubules at 1 day post fertilization (dpf). At 2dpf *clic5* transcripts were detected in the eye lenses, pectoral fin buds, liver primordium, and in the pronephric tubules and glomerulus. At 5dpf *clic5* expression was detectable in the liver and gut. The use of isoform specific *clic5* WISH probes revealed that *clic5a* was expressed in the eye lenses and pronephric glomerulus at 2dpf whereas *clic5b* was expressed in the pronephric tubules at 1dpf and additionally in the liver primordium at 2dpf. Given that *clic5b* is specifically expressed in the highly ciliated pronephric tubules, we next performed a triple whole-mount immunostaining on 1dpf old *Tg(actb2:Mmu.Arl13b-GFP)* embryos using antibodies for Clic5, GFP (labeling Arl13b-GFP, i.e. the ciliary axoneme) and γTubulin (labeling the ciliary basal body). These analyses revealed specific ciliary localization of Clic5, most presumably the Clic5 isoform Clic5b, in the pronephric tubules and surrounding tissue cells in zebrafish (Fig. 2, Suppl. Fig. 3).

### Knockdown of Clic5 leads to ciliopathy-associated phenotypes and leakiness of the glomerular filtration barrier in zebrafish

**Figure 3:**
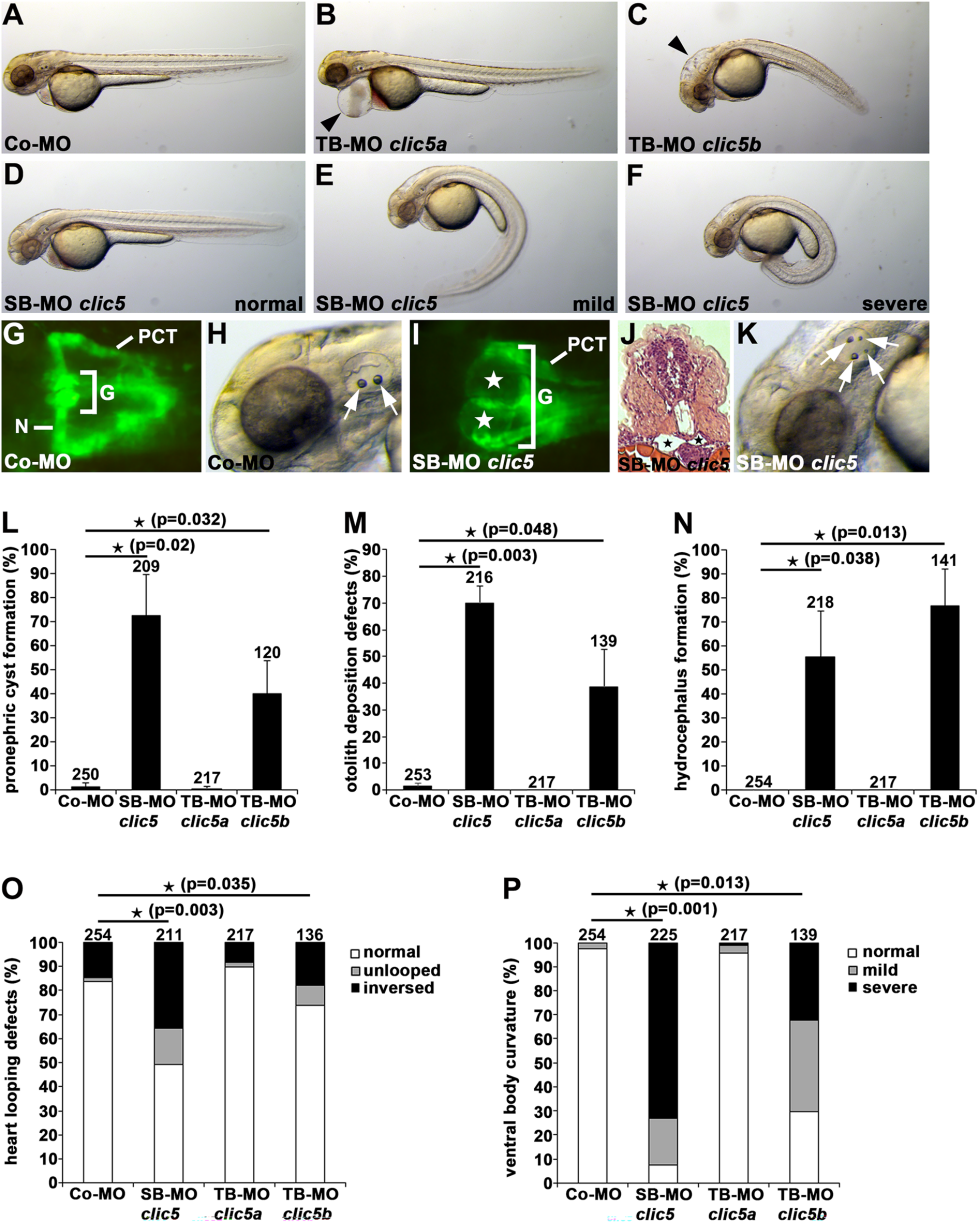
Clic5 knockdown analyses of cilia-related phenotypes in zebrafish. **(A–F)** Bright-field images of zebrafish embryos at 2dpf injected with Co-MO (6 ng) **(A)**, translation-blocking morpholino (TB-MO) *clic5a* (6 ng) **(B)**, TB-MO *clic5b* (4 ng) **(C)** and splicing-blocking morpholino (SB-MO) *clic5* (4 ng) **(D–F)**. In comparison to Co-MO injected embryos, injection of TB-MO *clic5a* leads to pericardial edema (black arrowhead) and injection of TB-MO *clic5b* or SB-MO *clic5* leads to hydrocephalus formation (black arrowhead) and different degrees of ventral body curvature. **(G–K)** Knockdown of Clic5 leads to pronephric cyst formation (white and black stars in (**I,J**), respectively) and otolith deposition defects (white arrows) at 2dpf as shown in a dorsal view with anterior to the left of a SB-MO *clic5* injected *Tg(wt1b:EGFP)* embryo (glomerulus (G), neck (N), proximal convoluted tubule (PCT)) **(I)**, and an embryo shown in a bright-field image **(K)**, respectively, in comparison to Co-MO injected embryos **(G,H)**; histological transverse section reveals pronephric cyst formation in a SB-MO *clic5* injected embryo **(J)**. **(L–P)** Quantification of pronephric cyst formation **(L)**, otolith deposition defects **(M)**, hydrocephalus formation **(N),** altered heart looping **(O)** and ventral body curvature **(P)** in 2dpf zebrafish embryos injected with Co-MO (6 ng), SB-MO *clic5* (4 ng), TB-MO *clic5a* (6 ng) or TB-MO *clic5b* (4 ng).

**Figure 4:**
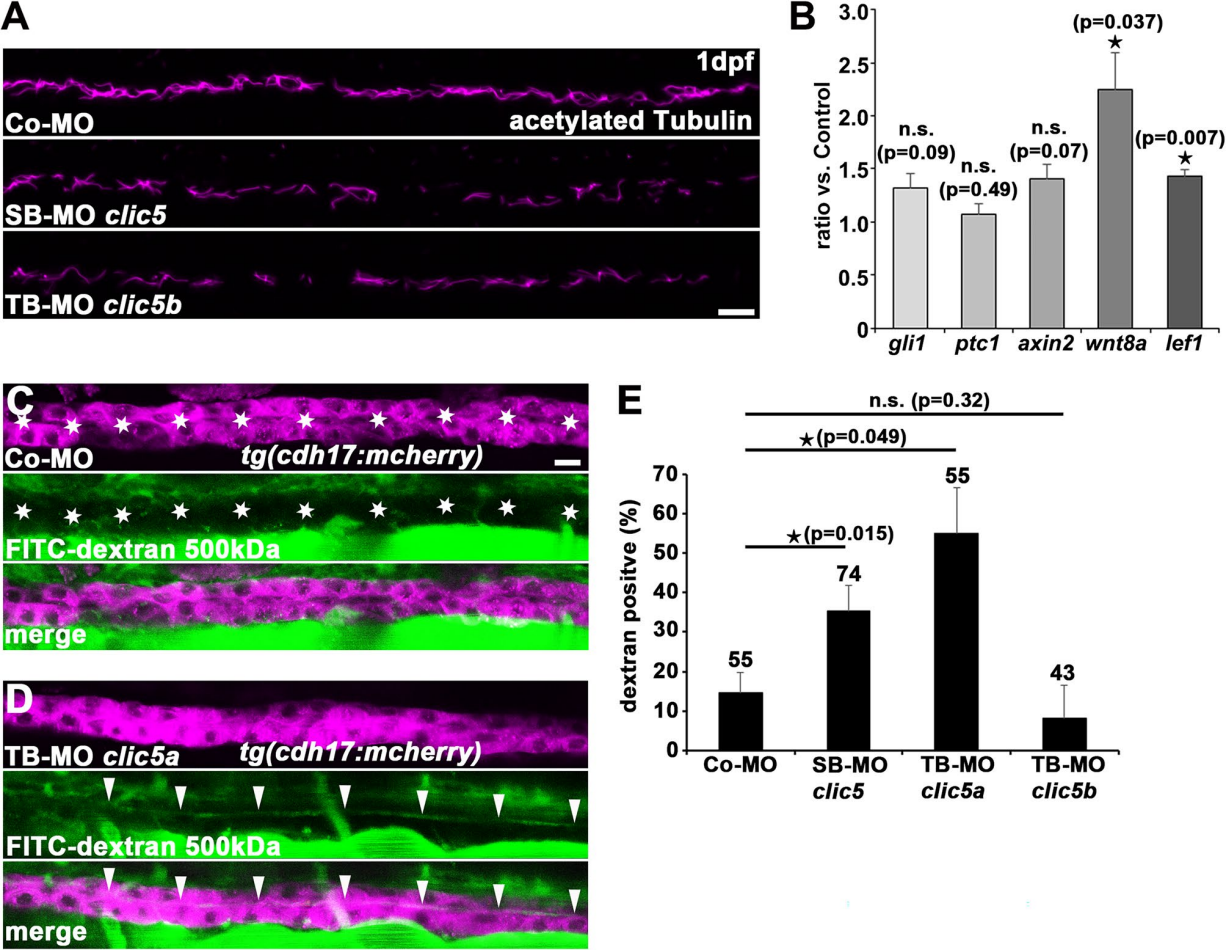
Analyses of Clic5 in ciliogenesis, and in ciliary and glomerular function in zebrafish. **(A)** Anti-acetylated Tubulin immunostaining reveals reduced cilia formation in the pronephric tubules of embryos injected with SB-MO *clic5* (4 ng) or TB-MO *clic5b* (4 ng) in comparison to Co-MO (4 ng) injected embryos at 1dpf. Representative confocal images depict the middle part of the pronephric tubule for each condition with anterior to the left. Scale bar: 10 µm. **(B)** Quantitative RT-PCR analyses reveals unaltered expression of Hedgehog signalling components *gli1* and *ptc1* while Wnt signalling components *axin2*, *wnt8a* and *lef1* were upregulated upon SB-MO *clic5* (4 ng) mediated knockdown compared to the control at 1dpf. **(C,D)** Injection of TB-MO *clic5a* (6 ng) leads to detectable FITC-dextran 500 kDa in the pronephric tubules at 4dpf of zebrafish development in comparison to Co-MO (6 ng) injected embryos shown by respective confocal images of *Tg(cdh17:mcherry)* embryos. Scale bar: 10 µm. **(E)** Quantification reveals statistic significant FITC-dextran 500 kDa positive embryos for the knockdown of Clic5 and Clic5a compared to the knockdown of Clic5b or the control.

We performed Morpholino (MO)-mediated knockdown experiments to analyse the specific function of Clic5 isoforms in zebrafish. For our experiments, we used a *clic5* splice-blocking (SB) MO targeting both *clic5* isoforms, and two translation-blocking (TB) MOs targeting the initiation codon of *clic5a* and *clic5b*, respectively (Suppl. Fig. 4). *Tg(wt1b:gfp)* embryos injected with SB-MO *clic5* or TB-MO *clic5b* revealed ciliopathy-associated phenotypes such as pronephric cyst formation, defective LR-asymmetry, ventral body curvature, otolith deposition defects and hydrocephalus formation. These phenotypes were not observed in embryos injected with Control-MO or TB-MO *clic5a* (Fig. 3). Co-injection of *clic5a*/*clic5b* mRNAs or *clic5b* mRNA partially prevented the observed phenotypes of Clic5 or Clic5b morphant embryos, respectively, confirming MO specificity (Suppl. Fig. 5). Analysis of cilia formation using anti-acetylated Tubulin immunostaining revealed reduced ciliogenesis in the pronephric tubules of Clic5 deficient embryos compared to the control at 1dpf (Fig. 4A). Moreover, we performed quantitative RT-PCR to analyse cilia-dependent signalling pathways in Clic5 deficient embryos at 1dpf. While Hedgehog signalling was unaffected, we observed dysregulation of Wnt signalling pathway components in Clic5 deficient embryos compared to the control (Fig. 4B). In summary, these results indicate a specific function for the Clic5 isoform Clic5b in cilia formation and/or function in zebrafish. Previous results identified Clic5a as a component of the Ezrin-Podocalyxin complex in glomeruli^23,24^. Loss of CLIC5A in mice leads to defective podocyte integrity and microalbumuria, and an individual affected with a homozygous bi-allelic CLIC5 mutation shows an elevated albumin/creatine ratio and pre-hypertension^23,24,28^. Knockdown of Clic5a in zebrafish resulted in prominent pericardial edema formation at 2dpf, a phenotype that has been described previously in zebrafish embryos deficient in important slit-diaphragm components such as Podocin and Nephrin (Fig. 3B)^30^. Co-injection of *clic5a* mRNA partially prevented pericardial edema formation of Clic5a morphant embryos, thus confirming MO specificity (Suppl. Fig. 5). We performed a well described permeability assay to analyse whether Clic5a has an essential function in the formation or maintenance of the glomerular filtration barrier, or both of these, in zebrafish^30,31^. Therefore, we injected the macromolecular 500 kDa lysine fixable fluorescein isothiocyanate (FITC) dextran into the common cardinal vein of 80 h post fertilization (hpf) *tg(cdh17:mcherry)* embryos that had been injected with Control-MO, SB-MO *clic5*, TB-MO *clic5a* or TB-MO *clic5b*. At 96hpf these embryos were subsequently assessed for the presence of FITC-dextran in the pronephric tubules. Quantification revealed that, compared to the control and Clic5b morphants, knockdown of Clic5 and Clic5a resulted in more FITC-dextran positive pronephric tubules (Fig. 4C–E). Taken together, these results indicate an essential role for Clic5a in the development and/or maintenance of a proper filtration barrier function in zebrafish.

### Knockdown of Clic5b results in decreased phosphorylated ERM levels in zebrafish

**Figure 5:**
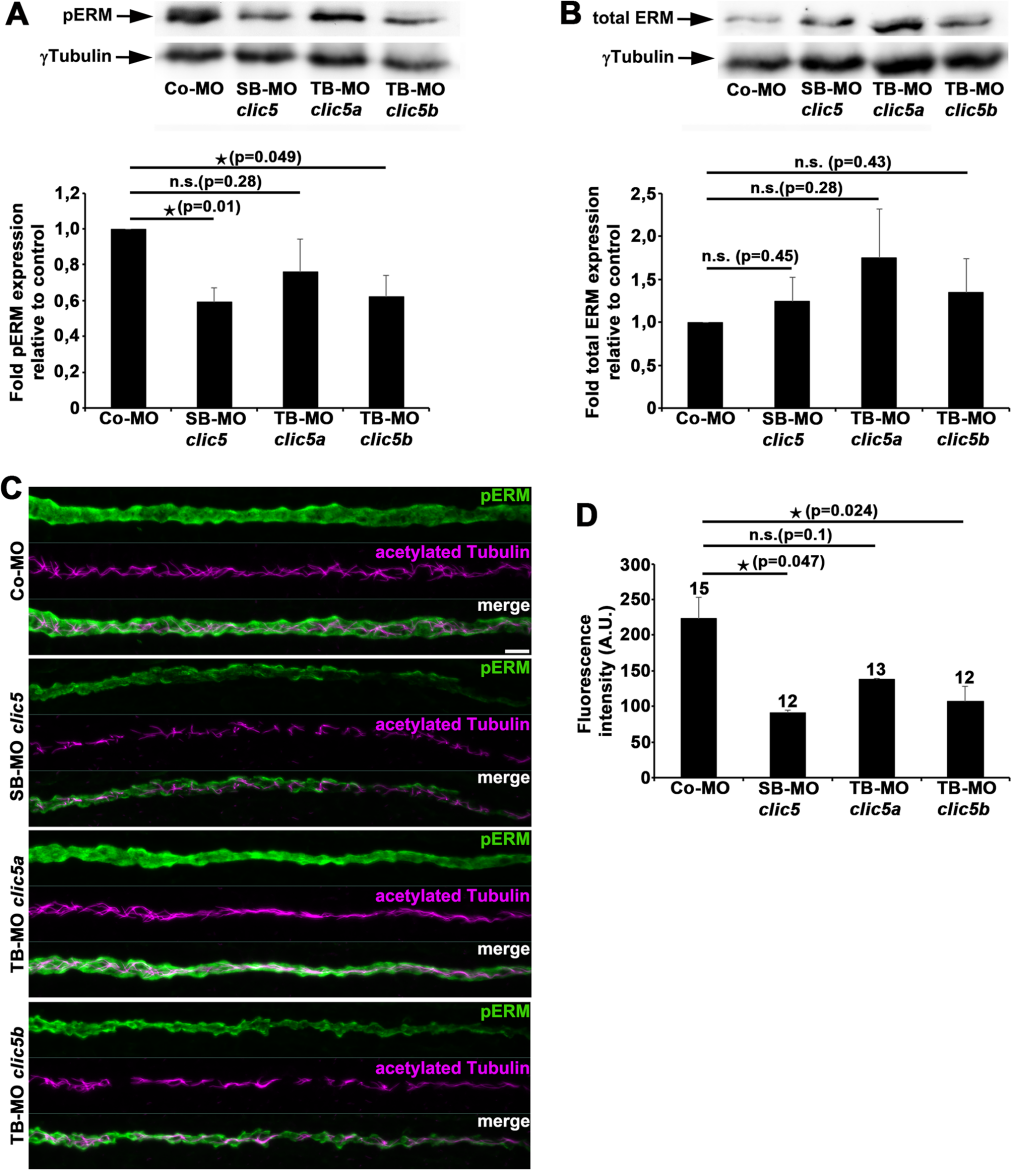
Analyses of pERM and total ERM levels after knockdown of Clic5, Clic5a or Clic5b. **(A)** Immunoblot analyses and respective quantification reveals significant decreased pERM levels in whole protein lysates from 1dpf old embryos that were injected with SB-MO *clic5* (2 ng) or TB-MO *clic5b* (2 ng) compared to TB-MO *clic5a* (3 ng) or Co-MO (3 ng) injected embryos. **(B)** Immunoblot analyses and respective quantification of total ERM levels reveals no significant difference in whole protein lysates from 1dpf old embryos that were injected with SB-MO *clic5* (2 ng), TB-MO *clic5a* (3 ng) or TB-MO *clic5b* (2 ng) compared with Co-MO (3 ng) injected embryos. **(C,D)** Double whole-mount immunostaining and respective quantification of 1dpf old zebrafish embryos using anti-pERM (green) and anti-acetylated Tubulin (magenta) antibodies reveals significant reduced pERM levels in the pronephric tubules of embryos injected with SB-MO *clic5* (2 ng) or TB-MO *clic5b* (2 ng) compared to TB-MO *clic5a* (3 ng) and Co-MO (3 ng) injected embryos; Arbitrary Unit (A.U.). Scale bar: 10 µm.

We next aimed to address mechanistic aspects of Clic5b function during ciliogenesis. Previous data indicates that CLIC5A co-localizes and interacts with ERM proteins and regulates ERM activity^16,22–27,32^. Our previous data revealed high expression of Ezrin in different ciliated tissues in zebrafish and its important role during ciliogenesis^33,34^. In addition, we identified the Elmo1-Dock180-Rac1 complex as a negative regulator of ERM activity^33^. We therefore speculated that Clic5b influences ERM activity during the process of ciliogenesis. Because only the *clic5b* isoform is specifically expressed in the pronephric tubules at 1dpf, we used whole embryo protein lysates from this stage for immunoblot analysis. Using either the SB-MO *clic5* or TB-MO *clic5b* resulted in significant decreased phosphorylated ERM (pERM) levels compared to respective lysates from Co-MO or TB-MO *clic5a* injected embryos. Moreover, analyses of total ERM levels revealed no significant difference in lysates of Clic5, Clic5a and Clic5b morphant embryos compared to the control (Fig. 5A,B). In addition, double whole-mount immunostainings on 1dpf old zebrafish embryos using antibodies for pERM and acetylated Tubulin revealed a significant reduction of pERM levels in the pronephric tubules of Clic5 and Clic5b morphant embryos in comparison to Clic5a morphants and the control (Fig. 5C,D). In summary, these data indicate that the Clic5b isoform is involved in the positive regulation of ERM activity during ciliogenesis.

## Discussion

To date the function of the CLIC family member CLIC5 is not completely understood. So far, most reports investigated the role of the smaller CLIC5 isoform CLIC5A. Apart from one study revealing non-selective ion conductance for CLIC5A in vitro, several studies in mice revealed important roles for CLIC5A in stereocilia organization of the inner ear and glomerular podocyte integrity^16,22–24,32^. In addition, previous reports demonstrated that CLIC5A co-localizes and/or interacts with ERM proteins and is a positive regulator of ERM activity that in turn act as linkers between the plasma membrane and the actin cytoskeleton^16,22–27,32^. CLIC5B co-localizes with AKAP350 at the Golgi apparatus and centrosomes, but a specific function for the longer CLIC5 isoform is still lacking^21^. One report identified both CLIC5 isoforms in the glomeruli of mice; glomerular CLIC5B expression was much lower than that of CLIC5A, but CLIC5B was much more abundant in a slit-diaphragm enriched fraction^23^. CLIC proteins CLIC1, CLIC5 and CLIC6 have been identified in different cilia-related proteomic analyses^35–39^. In addition, *CLIC3*, *CLIC5* and *CLIC6* were identified as transcriptionally regulated genes during mucociliary differentiation of primary human airway epithelial cells^40^. However, a ciliary role of CLIC proteins has not been described so far. Defects in cilia formation and/or function lead to a set of human related diseases, commonly referred to as ciliopathies that represent a major health burden. Despite of progress in identifying and characterizing cilia-related proteins in disease, a considerable number of ciliopathy-causing factors is still unknown or only poorly characterized.

In this study, we analysed the expression and role of Clic5 in zebrafish and identified a so far undescribed role for Clic5b in cilia formation and function. We identified *clic5a* expression in the eye lens and glomerulus, and Clic5a knockdown resulted in leakiness of the glomerular filtration barrier, thus confirming previous observations in CLIC5 knockout mice^23,24^. Notably, *clic5b* expression analysis revealed a distinct expression as *clic5a; clic5b* transcripts were detectable in the pectoral fin buds, liver, gut and the highly ciliated pronephric tubules in zebrafish. Immunostainings revealed ciliary localization of Clic5, most presumably Clic5b, in the pronephric tubules. Knockdown of Clic5b resulted in well-known ciliopathy-related phenotypes. In addition, our data demonstrated that cilia-dependent Wnt signalling is significantly disrupted in Clic5 deficient zebrafish embryos.The membrane-cytoskeletal linker Ezrin is highly expressed in ciliated tissues in zebrafish and previous data showed that Ezrin plays an important role during ciliogenesis in zebrafish and *Xenopus*^33,34^. In addition, we previously reported that inactivation of the bipartite guanine nucleotide exchange factor Elmo1/Dock180 or its downstream effector Rac1 resulted in increased ERM activation within the pronephric tubules in zebrafish embryos^33^. Our data now identified Clic5 as a positive regulator of ERM activity in the pronephric tubules. Therefore, Clic5 function on ERM activity during the process of ciliogenesis is in line to that reported for CLIC5 in cell culture systems and renal glomeruli in mice. If Clic5 acts within the Elmo1-Dock180-Rac1 complex remains to be determined.

## Materials and methods

### Zebrafish husbandry, lines and embryo maintenance

The fish used in this study were maintained at the Zebrafish Facility of the Medical Center of the University of Freiburg. All animal work has been conducted according to relevant national and international guidelines^41^. The study was approved by the Institutional Animal Care of the Medical Center of the University of Freiburg and the Regional Council Freiburg (permit ID G-16/89). All methods were carried out in accordance with ARRIVE guidelines. Zebrafish were maintained and embryos were staged as previously described^42^. The following strains were used: AB/TL wildtype, *Tg(wt1b:EGFP)*^43^ and *Tg(actb2:Mmu.Arl13b-GFP)*^44^. *Tg(cdh17:mcherry)* strain was generated by injection of a Tol2 vector^45^ containing a 4 kb fragment from the zebrafish cadherin17 promoter driving the expression of mCherry protein.

### Reverse-transcription polymerase chain reaction (RT-PCR) analysis

Semi-quantitative RT-PCR was performed to determine expression of zebrafish *clic5a* and *clic5b* during embryonic development and in adult organs. Total RNA from entire zebrafish embryos or adult zebrafish organs was extracted with the RNeasy Kit (Qiagen), followed by complementary DNA (cDNA) synthesis with the ProtoScript First Strand cDNA Synthesis Kit (Promega). Analysis of zebrafish *ef1α* was used as a loading control. The following primers were used for PCR analysis: *clic5a* (forward: 5ʹ-GGAGAGCACTAGATTCACCT-3ʹ, reverse: 5ʹ-TCTTGACCGTCTCCCATTGT-3ʹ); *clic5b* (forward: 5ʹ-TCCATCATGGCTGCAAACGT-3ʹ, reverse: 5ʹ-ACTGAAAGCCATGAGCAGGT-3ʹ); *ef1α* (forward: 5ʹ-ATCTACAAATGCGGTGGAAT-3ʹ; reverse: 5ʹ-ATACCAGCCTCAAACTCACC-3ʹ).

### Synthesis of antisense and sense RNA and in situ analysis

A zebrafish *clic5* probe detecting both *clic5* isoforms (pan probe) was amplified from zebrafish cDNA with primers (*clic5*, forward: 5ʹ-GGAGAGCACTAGATTCACCT-3ʹ, reverse: 5ʹ-TTACTTGGTTAGCCTCTTGG-3ʹ). The N-terminal part of zebrafish *clic5a* and *clic5b* were amplified from zebrafish cDNA with specific primers (*clic5a*, forward: 5ʹ-GGAGAGCACTAGATTCACCT-3ʹ, reverse: 5ʹ-TCTTGACCGTCTCCCATTGT-3ʹ; *clic5b* forward: 5ʹ-TCCATCATGGCTGCAAACGT-3ʹ, reverse: 5ʹ-ACTGAAAGCCATGAGCAGGT-3ʹ). Amplification products were cloned into TOPO (Invitrogen), followed by sequence verification and linearization with corresponding restriction enzymes for synthesis of antisense or sense RNA. Whole-mount in situ hybridization (WISH) analysis using Digoxigenin-labelled probes was performed as described^46^ using NBT (blue) (Roche) as substrate.

### Immunofluorescence, immunoblotting and histology

Zebrafish whole-mount immunofluorescence (IF) was performed as previously described^33^. The following antibodies were used for IF: anti-CLIC5 (B-23; sc-133468; Santa Cruz Biotechnology; 1:10), anti-CLIC5 (A-11; sc-271863; Santa Cruz Biotechnology; 1:10), anti-γTubulin (clone GTU‐88; Sigma Aldrich; 1:5000), anti-GFP (ab13970; Abcam; 1:200), anti-acetylated αTubulin (clone 6-11B-1; Sigma Aldrich; 1:500) and anti-phospho-Ezrin (Thr567) (PA5-37763; Invitrogen; 1:200). Cy3 (1:1000) and Alexa-488/-546/-647 (1:1000) labelled secondary antibodies were purchased from Jackson Immunoresearch and Molecular Probes (Invitrogen), respectively. Immunoblotting (IB) was performed as previously described^47^. The following antibodies were used for IB: anti-CLIC5 (B-23; sc-133468; Santa Cruz Biotechnology; 1:200), anti‐pERM (#3141; Cell Signaling; 1:1000), anti‐ERM (#3142; Cell Signaling; 1:1000), anti‐γTubulin (clone GTU‐88; Sigma Aldrich; 1:5000) and respective HRP‐conjugated antibodies (DAKO; 1:5000). Histological and transverse sectioning procedures were performed as previously described^33^.

### mRNA and morpholino (MO) injection

For synthesis of mRNA, we used full-length zebrafish clic5a-pCS2+ and clic5b-pCS2+. mRNA was prepared from Acc651-linearized clic5a-pCS2+, KpnI-linearized clic5b-pCS2+ using SP6 mMessage mMachine Kit (Ambion). Morpholino oligonucleotide (MO) injection was performed as described^33^. To attenuate possible off target effects, a p53 MO^48^ was coinjected 1.5-fold to the other MOs used. The following Translation/Splicing-Blocking (TB/SB) antisense MOs (Gene Tools) were used for zebrafish: SB-MO *clic5* 5ʹ-CCAGCCTACAAACACAACCACACAT-3ʹ; TB-MO *clic5a* 5ʹ-CTTGACCGTCTCCCATTGTAGACAC-3ʹ; TB-MO *clic5b* 5ʹ-GACGTTTGCAGCCATGATGGACCTC-3ʹ; and a Standard Control (Co)-MO 5ʹ-CCTCTTACCTCAGTTACAATTTATA-3ʹ. The Co-MO is thought to have no target and only very little biological activity (https://www.gene-tools.com/custom_morpholinos_controls_endmodifications). The TNT Quick Coupled Transcription/Translation System (Promega) was used to confirm the MO efficiency for TB-MO *clic5a* and TB-MO *clic5b* with full-length zebrafish *clic5a* (clic5a-pCS2+) and *clic5b* (clic5b-pCS2+), respectively.

### Quantitative real-time PCR (qPCR)

Total RNA was obtained from 30 Co-MO or SB-MO *clic5* injected zebrafish embryos at 1dpf using the RNeasy Kit (Qiagen). First strand cDNA synthesis was performed using the cDNA Reliance Select Synthesis Kit (BIO-RAD). qPCR was performed on a Light Cycler 480 (Roche) using the Blue S’Green Master Mix (Biozym). 10 µl reaction volumes were used with following cycle program: 95 °C for 2 min (95 °C for 5 s, 60 °C for 30 s) × 44. *actb1* and *ef1α* were used as normalization control. Technical triplicates of four biological samples were analysed for gene expression. Melt curve analysis was performed. The following primers were used for qPCR analysis: *actb1* (forward: 5ʹ-CCTTCCTTCCTGGGTATGG-3ʹ, reverse: 5ʹ-GGTCCTTACGGATGTCCAC-3ʹ); *ef1α* (forward: 5ʹ-TGCCAACTTCAACGCTCAGGTC-3ʹ, reverse: 5ʹ-TCAGCAAACTTGCAGGCGATG-3ʹ); *gli1* (forward: 5ʹ-TCAGACGTCCTCTCGCCTTA-3ʹ, reverse: 5ʹ-AGCTCATGTCTCCGATTGCC-3ʹ); *ptc1* (forward: 5ʹ-GGGTCCTGAATGGACTGGTG-3ʹ, reverse: 5ʹ-CCGCTGGAGATACCTCAGGA-3ʹ); *axin2* (forward: 5ʹ-ACCCTCGGACACTTCAAGGA-3ʹ, reverse: 5ʹ-GTGCAGTCATCCCAGACCTC-3ʹ); *wnt8a* (forward: 5ʹ-ATTCGTGGATGCGCTTGAGA-3ʹ, reverse: 5ʹ-TTACAGCCAAACGTCCAGCTT-3ʹ); *lef1* (forward: 5ʹ-CAGACATTCCCAATTTCTATCC-3ʹ, reverse: 5ʹ-TGTGATGTGAGAACCAACC-3ʹ).

### Fluorescent dye injection

Lysine fixable fluorescein isothiocyanate (FITC) conjugated dextran 500 kDa (Molecular Probes; 25 mg/mL diluted 1:20 in H_2_O) was injected into the common cardinal vein (CCV) of MO-injected 80hpf embryos, anesthetized with 0.4% tricaine in Danieau’s solution. Before injection of the fluorescent tracer, a sufficient blood circulation was checked by eye, judged by enough moving red blood cells. Afterwards the embryos were transferred to fresh Danieau’s solution with PTU for overnight incubation at 28.5 °C. At 96hpf embryos were checked again for proper blood circulation and subsequently analysed at the scanning confocal microscope for the presence of 500 kDa Dextran in the pronephric tubules.

### Microscopy, image acquisition, and statistical analysis of immunofluorescence

Confocal imaging of zebrafish embryos was performed using confocal microscopes LSM510 and LSM980 ZEISS (ZEISS objectives: Achroplan NIR 40×/0.8 water-immersion and Plan-Apochromat 40×/1.0 DIC VIS-IR water-immersion, respectively). Zebrafish embryos were embedded in 1% low-temperature melting agarose (Biozym) in 30% Danieau’s solution. Vertical projections of recorded stacks were generated using LSM Examiner (ZEISS) or Imaris software (Oxford Instruments). Proteins in maximum projection Z-stacks were segmented using both the surface (Arl13b-GFP, γTubulin) and Particle (Clic5) extensions of the IMARIS 9.9.1 software. To determine protein co-localization, Clic5 voxels were analysed for their presence inside Arl13-GFP or γTubulin surfaces, respectively. Brightfield images of whole-mount in situ embryo stains were taken using an Axioplan2 microscope with Axiocam camera and using Axiovision software (ZEISS). Embryos of the *Tg(wt1b:EGFP)* line were analysed under a Leica MZ16 stereo-microscope (Leica, Solms, Germany), and non-confocal fluorescent images were taken with a SPOT Insight Fire Wire System (Diagnostic Instruments, Sterling Heights, MI). All images were exported as TIFF files and imported into Adobe Photoshop software CS2 to arrange figures. For the quantification of pERM fluorescence intensity, a region of interest (ROI), which included the anterior part of the pronephric tubule, was selected. All intensity measurements were performed using ImageJ Fiji (https://fiji.sc/).

### Statistical analysis and quantification

All data represent results from at least one of three independent experiments with similar results. Numbers of embryos used for analysis are indicated in the respective bar chart unless otherwise stated. Data were analysed by Student’s *t*-test (2-sided, unpaired); error bars in Figs. 3, 5A,B and Suppl. Fig. 5 represent the standard deviation (SD); error bars in Figs. 4E and 5D represent the standard error of the mean (SEM). qPCR data were analysed with Graphpad Prism software and one sample *t*-test; error bars represent the SEM.

### Supplementary Information

Supplementary Figures.

## Data Availability

The data presented in this study are available on request from the corresponding author.
