# Supplementary material for: A novel role for the chloride intracellular channel protein Clic5 in ciliary function
Source: Sci Rep. 2023 Oct 17;13:17647. doi: 10.1038/s41598-023-44235-y (PMC10582032; doi:10.1038/s41598-023-44235-y)
Supplement: Supplementary file 1 — Supplementary Figures. [file 41598_2023_44235_MOESM1_ESM.docx]

**Supplementary Information**

**A novel role for the chloride intracellular channel protein Clic5 in ciliary function**

**Elisabeth Ott, Sylvia Hoff, Lara Indorf, Franck Anicet Ditengou, Julius Müller, Gina Renschler, Soeren S. Lienkamp, Albrecht Kramer-Zucker, Carsten Bergmann and Daniel Epting**

**Suppl. Fig. 1 Evolutionary conservation of CLIC family members.**

**Suppl. Fig. 2 Analyses of whole-mount *in situ* hybridization efficiency.**

**Suppl. Fig. 3 Subcellular localization analyses of Clic5 in the pronephric tubule and surrounding tissue cells of zebrafish.**

**Suppl. Fig. 4 Clic5 knockdown strategy and analyses of Clic5 knockdown efficiency.**

**Suppl. Fig. 5 Analyses of Morpholino specificity.**

**Suppl. Fig. 6 Respective uncropped and unprocessed gel images and immunoblots.**

**Suppl. Fig. 7 Respective uncropped and unprocessed immunoblot.**

**Supplementary Figure 1**

**
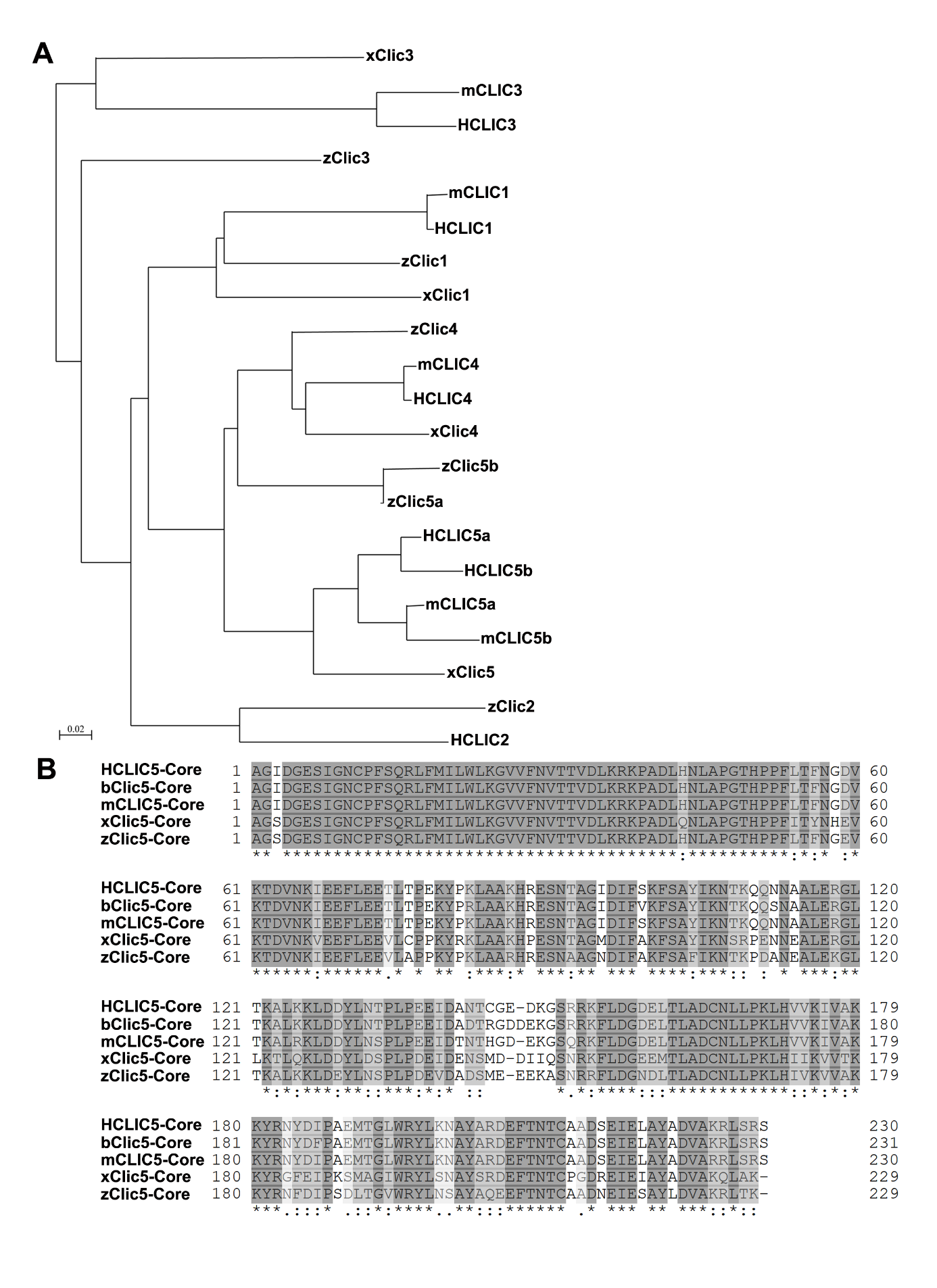
**

**Supplementary Figure 2**

**
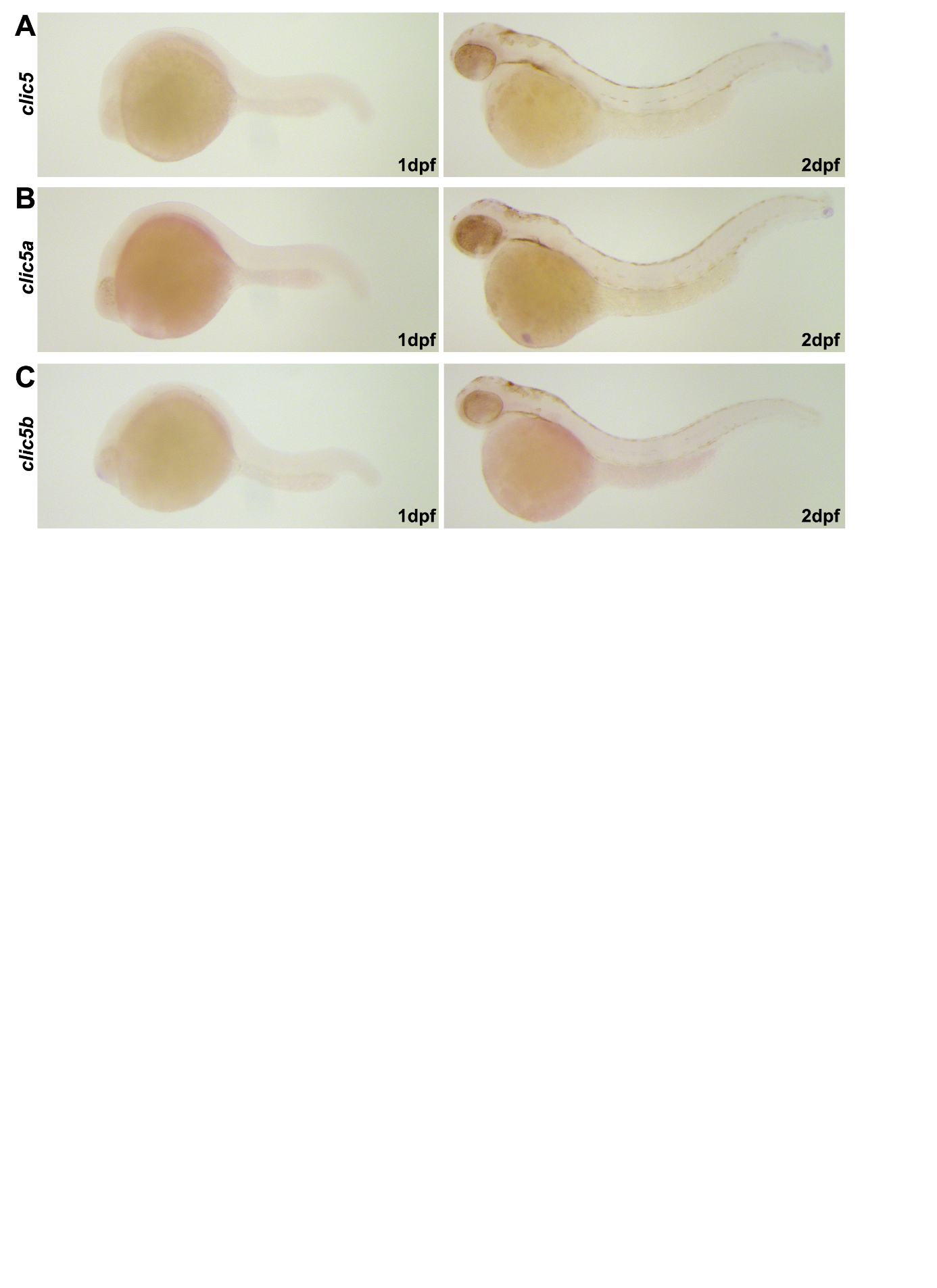
**

**Supplementary Figure 3**

**
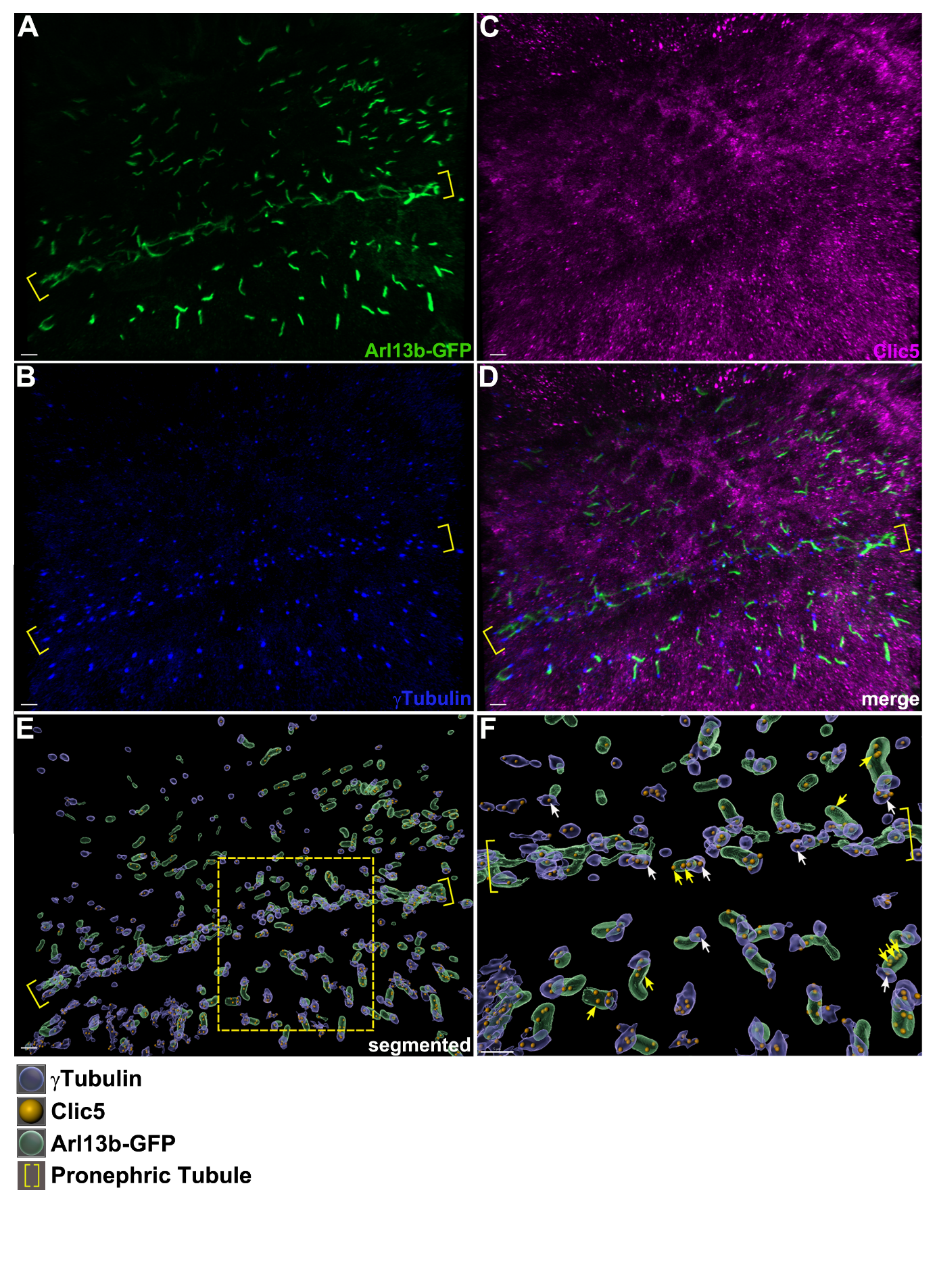
**

**Supplementary Figure 4**

**
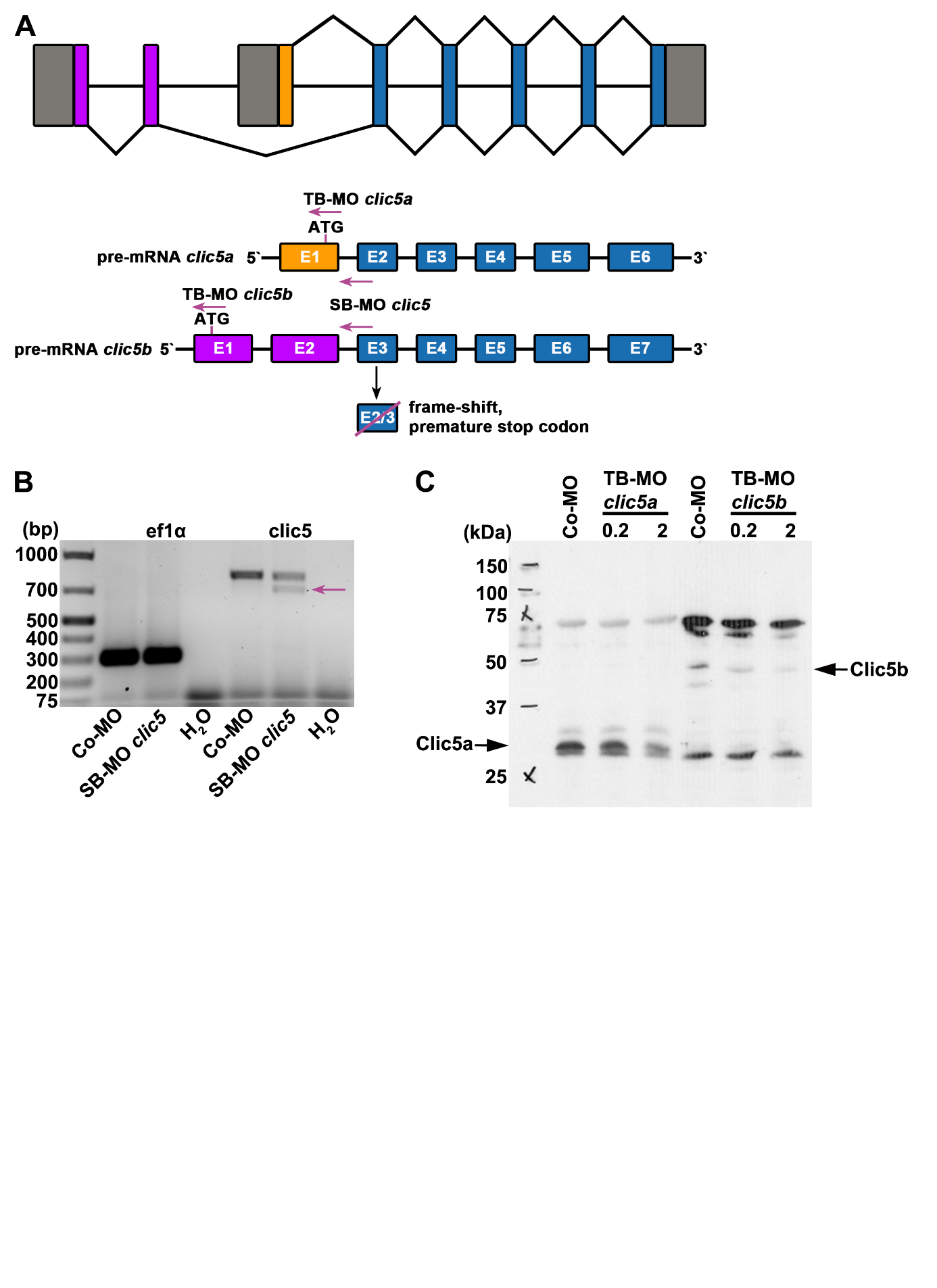
**

**Supplementary Figure 5**

**
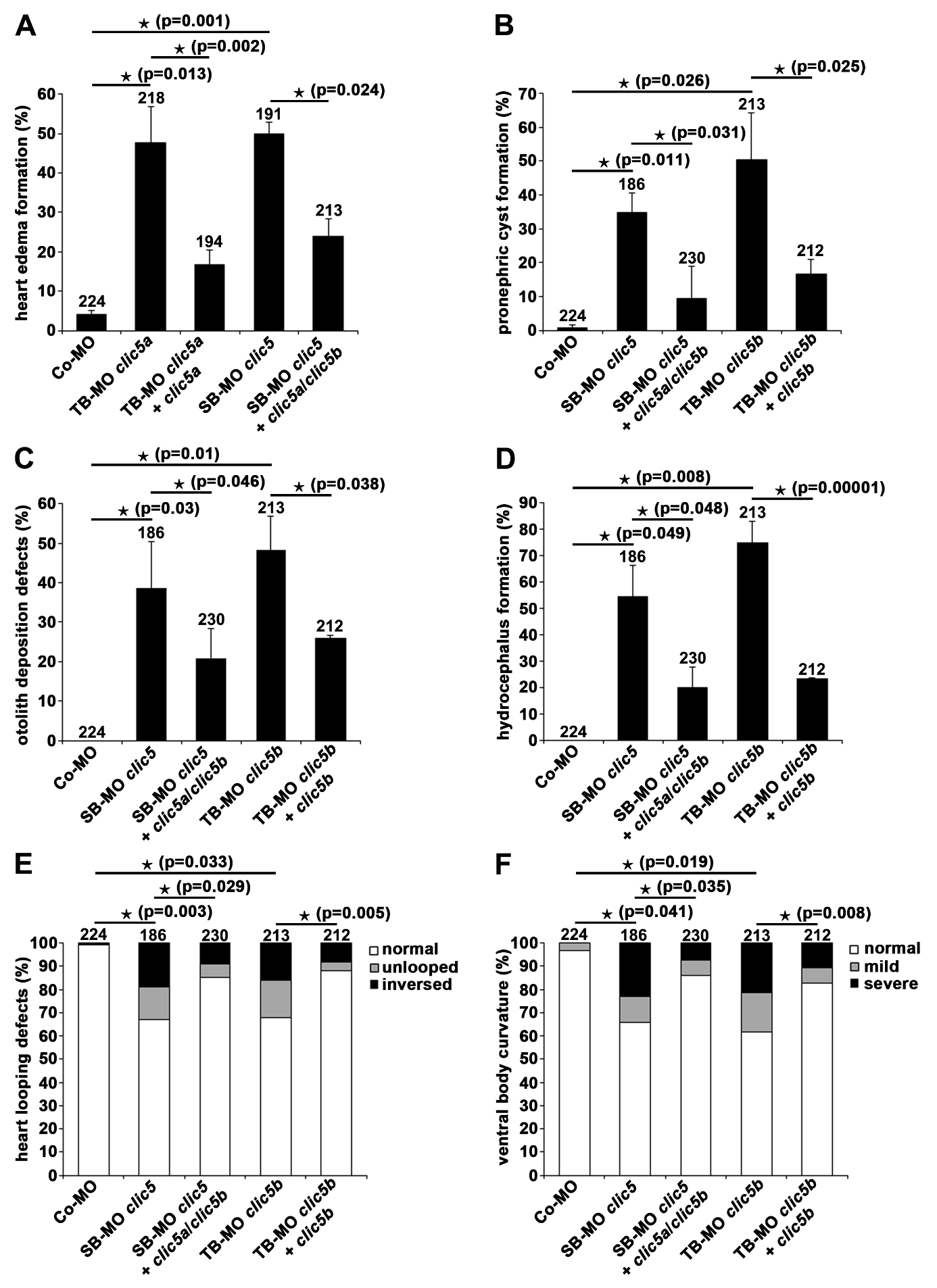
**

**Supplementary Figure 6**

**
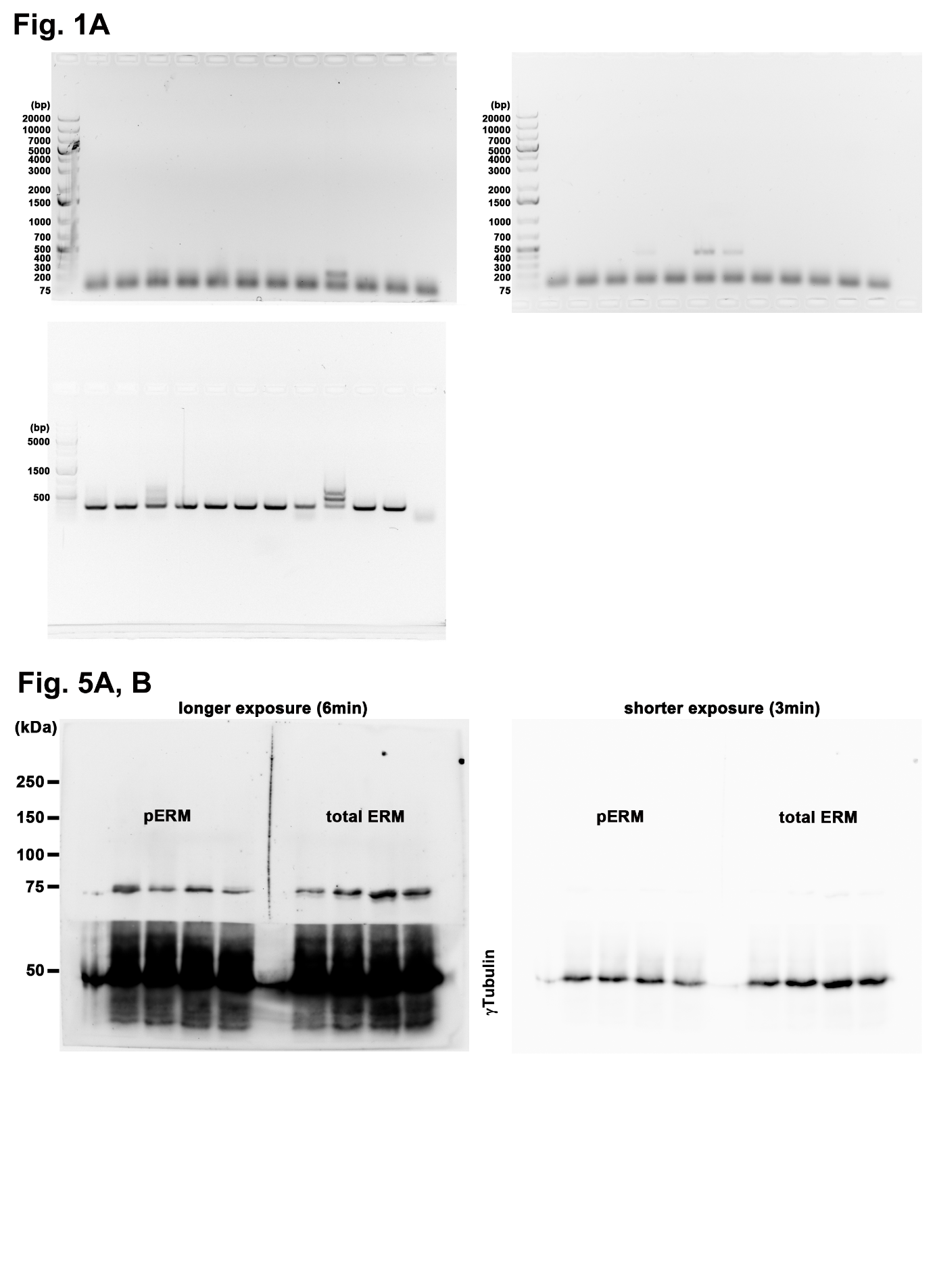
**

**Supplementary Figure 7**

**
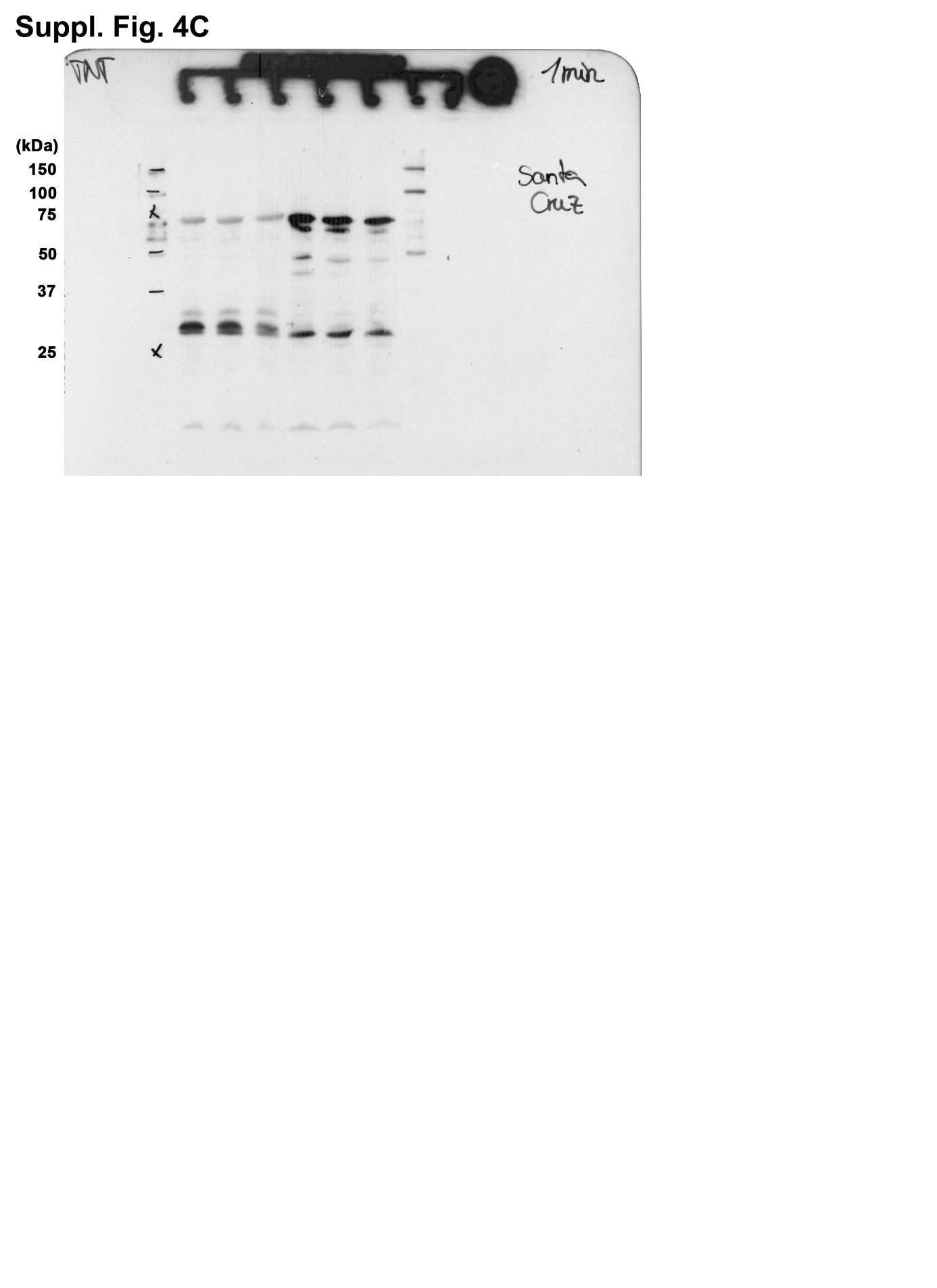
**

**Figure Legends**

**Suppl. Fig. 1 Evolutionary conservation of CLIC family members.**

**(A)** [Phylogenetic analysis](https://www.sciencedirect.com/topics/biochemistry-genetics-and-molecular-biology/phylogeny) of CLIC(1-5) family members from different vertebrates. Zebrafish Clic5 isoforms show the highest homology to the members of the CLIC4 and CLIC5 family. Scale bar indicates evolutionary distance. Accession codes: human (HCLIC1: NP_001274523.1, HCLIC2: NP_001280.3, HCLIC3: NP_004660.2, HCLIC4: NP_039234.1, HCLIC5A: NP_058625.2, HCLIC5B: NP_001107558.1); mouse (mCLIC1: NP_254279.1, mCLIC3: NP_081361.1, mCLIC4: NP_038913.1, mCLIC5A: NP_766209.1, mCLIC5B: XP_006524198.1); *Xenopus* (xClic1: AAI06697.1, xClic3: NP_001086217.1, xClic4: AAH72787.1) and zebrafish (zClic1: NP_997847.1, zClic2: NP_001002561.1, zClic3: NP_955818.1, zClic4: NP_958894.1, zClic5a: AAI62210.1, zClic5b: AAI55313.1). **(B)** Multiple amino acid alignment of solely the CLIC5 core domain from human CLIC5 (HCLIC5-Core), mouse CLIC5 (mCLIC5-Core), *Xenopus* Clic5 (xClic5-Core), cow Clic5 (bClic5-Core) and zebrafish Clic5 (zClic5-Core). Identical and similar amino acids are indicated by dark and light gray backgrounds, respectively.

**Suppl. Fig. 2 Analyses of whole-mount *in situ* hybridization efficiency.**

**(A-C)** Whole-mount *in situ* hybridization (WISH) analyses using respective sense RNA Digoxigenin-labelled probes (negative controls) result in no specific staining for *clic5* **(A)**, *clic5a* **(B)** or *clic5b* **(C)** at 1 and 2dpf.

**Suppl. Fig. 3 Subcellular localization analyses of Clic5 in the pronephric tubule and surrounding tissue cells of zebrafish.**

**(A-D)** Triple whole-mount immunostaining on 1dpf old *T*g(actb2:Mmu.Arl13b-GFP) embryos using anti-GFP (labeling Arl13b-GFP, i.e. the ciliary axoneme) **(A)**, anti-γTubulin (labeling the ciliary basal body) **(B)** and anti-Clic5 (polyclonal antibody produced in rabbit) **(C)** antibodies reveals ciliary localization of Clic5 in the pronephric tubule and surrounding tissue cells. **(D)** Image is **(A-C)** merged. **(E)** 3D reconstruction and segmentation of Arl13b-GFP (green), γTubulin (magenta) and Clic5 (gold) using Imaris software. **(F)** Image reflects a higher magnification of boxed area in **(E)**. In the pronephric tubule (yellow brackets) and surrounding tissue cells Clic5 co-localizes with Arl13b-GFP and γTubulin, demonstrating ciliary axoneme and ciliary basal body localization of Clic5, respectively. Clic5 voxels co-localize with those of ciliary basal body (white arrows) and ciliary axoneme voxels (yellow arrows), respectively. Scale bar, 5 µm.

**Suppl. Fig. 4 Clic5 knockdown strategy and analyses of Clic5 knockdown efficiency.**

**(A)** Exon structure and splicing scheme of two clic5 isoforms in zebrafish, referred as clic5a and clic5b (not drawn to scale). Translation start codons (ATG) and TB-MO *clic5a* and TB-MO *clic5b* are indicated. Expression silencing of clic5 using SB-MO clic5 leads to the exclusion of exon2 or exon3 of clic5a or clic5b, respectively, that results in a frameshift and premature stop codon of Clic5a and Clic5b. **(B)** RT-PCR reveals efficiency of the SB-MO clic5. The upper signal represents the wild-type splice product, whereas the lower signal (magenta arrow) represents the morphant exon-skipping product. H_2_O served as negative control and ef1α as loading control. **(C)** *In vitro* translated zebrafish Clic5a or Clic5b protein was detected via immunoblotting by CLIC5 antibody. Clic5a and Clic5b protein level decreased in the presence of 0.2μM and 2μM of the TB-MO *clic5a* or TB-MO *clic5b*, respectively.

**Suppl. Fig. 5 Analyses of Morpholino specificity.**

**(A)** Quantification of pericardial edema formation in 2dpf zebrafish embryos injected with Co-MO (6ng), TB-MO *clic5a* (6ng), TB-MO *clic5a* (6ng) + *clic5a* mRNA (2,5pg), SB-MO *clic5* (4ng) and SB-MO *clic5* (4ng) + *clic5a*/*clic5b* mRNAs (each 2,5pg). Co-injection of *clic5a* mRNA and *clic5a*/*clic5b* mRNAs results in significant prevention of pericardial edema formation in Clic5a and Clic5 morphant embryos, respectively. **(B-F)** Quantification of pronephric cyst formation **(B)**, otolith deposition defects **(C)**, hydrocephalus formation **(D)** altered heart looping **(E)** and ventral body curvature **(F)** in 2dpf zebrafish embryos injected with Co-MO (4ng), SB-MO *clic5* (4ng), SB-MO *clic5* (4ng) + *clic5a*/*clic5b* mRNAs (each 2,5pg), TB-MO *clic5b* (4ng) and TB-MO *clic5b* (4ng) + *clic5b* mRNA (2,5pg). Co-injection of *clic5a*/*clic5b* mRNAs and *clic5b* mRNA results in significant prevention of all analysed ciliopathy-associated phenotypes in Clic5 and Clic5b morphant embryos, respectively.

**Suppl. Fig. 6 Respective uncropped and unprocessed gel images and immunoblots.**

**Suppl. Fig. 7 Respective uncropped and unprocessed immunoblot.**
